# The Tree(s) of Life: The Human Placenta and My Journey to Learn More about It

**DOI:** 10.1371/journal.ppat.1005515

**Published:** 2016-04-21

**Authors:** Carolyn B. Coyne

**Affiliations:** Department of Microbiology and Molecular Genetics, Department of Obstetrics and Gynecology, Magee Women’s Research Institute, University of Pittsburgh School of Medicine, Pittsburgh, Pennsylvania, United States of America; The Fox Chase Cancer Center, UNITED STATES

I have always been fascinated by the mechanisms by which viruses, and other microorganisms, bypass and/or cross cellular barriers to enter their human hosts. As a trainee and young investigator, my research primarily focused on how enteroviruses cross the barriers presented by the epithelium and endothelium. Yet, despite all my years focused on cellular barriers, there was a barrier I had always overlooked, until faced with the stark reality of its importance—the placenta, which forms the barrier between mother and her developing baby during pregnancy.

Early in my pregnancy, as I sat prepping large volumes of highly infectious enteroviruses, a question suddenly occurred to me: “What is protecting my baby from these viruses?” A quick search revealed that we know shockingly little about how the human placenta shields fetuses from microorganisms and the mechanisms by which pathogens associated with fetal infections gain access to the fetal compartment. As a scientist and a pregnant woman, I was startled and, frankly, a bit alarmed.

Why do we know so little about the human placenta? I have learned that the answer to this very simple question is quite complex. First, there is a lack of suitable and/or easily accessible cell-based and small animal-based models that fully recapitulate the cellular and structural complexity of the human placenta. Furthermore, and rather reassuringly, the placenta is so effective at protecting and nourishing the developing fetus that its critical role in both processes is often overlooked and underappreciated. Finally, issues related to women’s health, particularly reproductive health, are often understudied relative to issues impacting the health of men. Collectively, these issues, coupled with others, have limited our understanding of many of the most basic aspects of how the placenta shields the fetus from microorganisms.

The placenta is an amazing and highly unique organ that has nurtured and protected us all. A common misconception is that the placenta is of maternal origin when, in fact, it develops from the fetus. Placentation, the process of placenta development, is initiated at the time of implantation, when the blastocyst first contacts the maternal endometrium. During these early stages of pregnancy, the placenta is responsible for both anchoring the blastocyst into the endometrium and for establishing the essential maternal and fetal circulatory systems. This process is amazingly complex, and defects at any stage can lead to miscarriage. Once developed, the placenta serves as the sole conduit between the mother and fetus and facilitates nutrient, gas, and waste exchange throughout pregnancy. In addition, the placenta acts as the key barrier that prevents the vertical transmission of pathogens from mother to fetus, as anything entering the fetal compartment must directly cross the placenta. In humans, this is a major task, as the placenta is bathed in ~150 mL of maternal blood in the second and third trimesters, which is delivered by as many as 100 spiral arteries in the final stages of pregnancy. Thus, much like young coral polyps or anemones in a reef, the villous tree surfaces of the human placenta are gracefully flowing with the current of maternal blood that is providing nutrients but also bathing the villous trees with pathogens present in the maternal blood.

Several pathogens can circumnavigate the placental barrier and enter the fetal compartment, often inducing devastating consequences. Perinatal infections are associated with ~2%–3% of congenital anomalies, most of which are caused by the “TORCH” pathogens, which include *Toxoplasma gondii*, Other (syphilis, varicella-zoster, parvovirus B19), Rubella, Cytomegalovirus (CMV), and Herpesviruses. However, the list of pathogens capable of inducing fetal disease continues to grow, as evidenced by the ongoing outbreak of Zika virus in Brazil, which has been associated with miscarriage and/or birth defects, including microcephaly. Several governments have gone so far as to encourage women to avoid becoming pregnant until we know more about how this virus induces fetal disease. However, as with most viruses, Zika virus is unlikely to remain contained in Brazil and has already been detected in several other countries, including the United States.

For me, as for most women, pregnancy was both an exciting and terrifying experience. As a virologist, I was acutely aware of the viruses in the air that I breathed and lying in wait on the surfaces that I touched. Perhaps inspiration can come to scientists as it does to artists—from diverse and sometimes unusual sources. Being pregnant with my son inspired me to learn about the placenta growing inside me and opened my eyes to the importance of this exceptional organ. It is imperative that we understand how existing and emerging pathogens associated with fetal disease breach the placental barrier. Only then will we develop medical treatments to prevent the vertical transmission of pathogens and protect our growing babies from the constant onslaught of microbes that infect mothers throughout pregnancy.

**Image 1:**
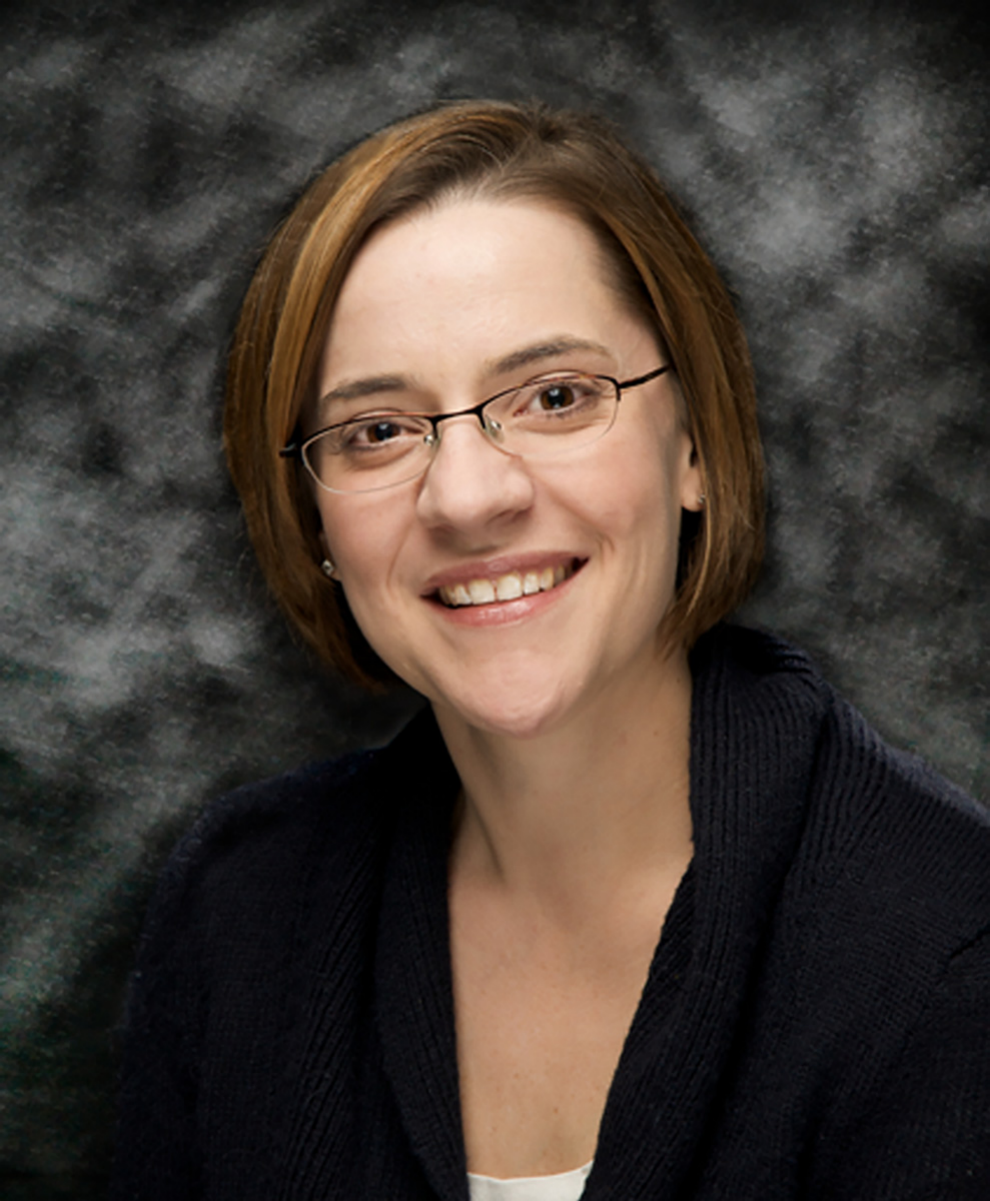
Carolyn B. Coyne.

**Image 2:**
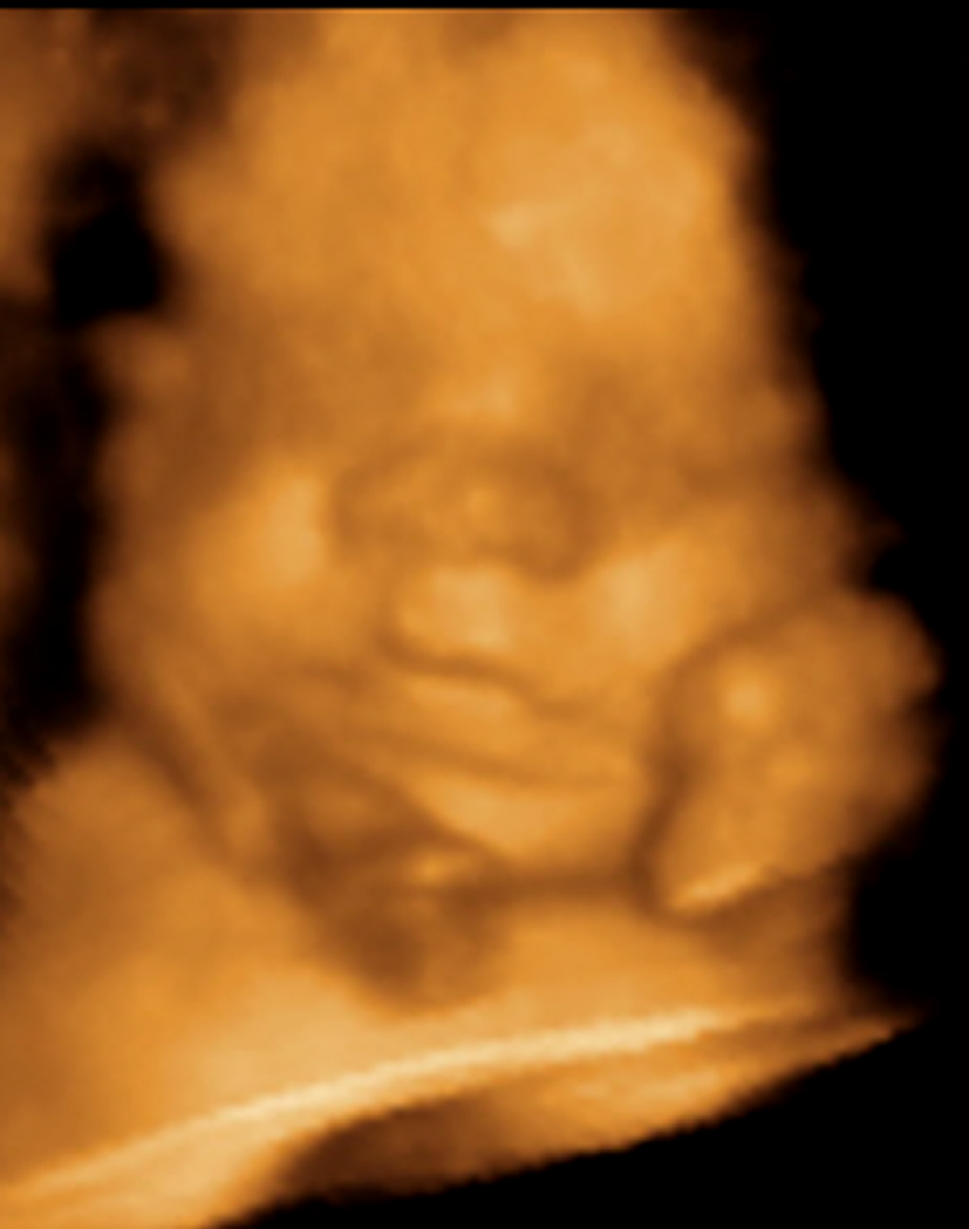
A three-dimensional ultrasound image of my son at ~30 weeks gestation, perhaps marveling at the placenta that protects him.

